# The Effect of Chronic Alprazolam Intake on Memory, Attention, and Psychomotor Performance in Healthy Human Male Volunteers

**DOI:** 10.1155/2016/3730940

**Published:** 2016-07-04

**Authors:** Zahid Sadek Chowdhury, Mohammed Monzur Morshed, Mohammad Shahriar, Mohiuddin Ahmed Bhuiyan, Sardar Mohd. Ashraful Islam, Muhammad Shahdaat Bin Sayeed

**Affiliations:** ^1^Department of Pharmacy, University of Asia Pacific, Dhaka 1209, Bangladesh; ^2^Department of Biochemistry and Molecular Biology, University of Dhaka, Dhaka 1000, Bangladesh; ^3^Department of Clinical Pharmacy and Pharmacology, University of Dhaka, Dhaka 1000, Bangladesh

## Abstract

Alprazolam is used as an anxiolytic drug for generalized anxiety disorder and it has been reported to produce sedation and anterograde amnesia. In the current study, we randomly divided 26 healthy male volunteers into two groups: one group taking alprazolam 0.5 mg and the other taking placebo daily for two weeks. We utilized the Cambridge Neuropsychological Test Automated Battery (CANTAB) software to assess the chronic effect of alprazolam. We selected Paired Associates Learning (PAL) and Delayed Matching to Sample (DMS) tests for memory, Rapid Visual Information Processing (RVP) for attention, and Choice Reaction Time (CRT) for psychomotor performance twice: before starting the treatment and after the completion of the treatment. We found statistically significant impairment of visual memory in one parameter of PAL and three parameters of DMS in alprazolam group. The PAL mean trial to success and total correct matching in 0-second delay, 4-second delay, and all delay situation of DMS were impaired in alprazolam group. RVP total hits after two weeks of alprazolam treatment were improved in alprazolam group. But such differences were not observed in placebo group. In our study, we found that chronic administration of alprazolam affects memory but attentive and psychomotor performance remained unaffected.

## 1. Introduction

There is a steadily increased rate of alprazolam prescriptions in Bangladesh, which has a population of more than 168 million (IMSQ4, 2015, in-house data) [1]. Generally, alprazolam is the most frequently used benzodiazepine [2] primarily indicated for the treatment of panic disorder and generalized anxiety disorder (GAD) [3, 4]. The clinical dose for the management of anxiety can range from 0.5 to 4 milligrams (mg) per day and a daily dose of up to 10 mg is indicated for the management of panic disorder [5]. Alprazolam has been shown to be as equally effective in the treatment of GAD as other benzodiazepines [6, 7], tricyclic antidepressants [8, 9], and serotonin reuptake inhibitors [10]. Alprazolam is also effective in the treatment of severe anxiety in patients during alcohol withdrawal [11]. It is also affective in the treatment of major depressive disorder when prescribed in double doses that are used for anxiety relief [12]. However, alprazolam is not a preferred choice in the treatment of body dysmorphic disorders. Alprazolam is considered as a preferred anxiolytic because of its additional role as an antidepressant [13]. Another benefit of this drug is the relatively faster onset of anxiety relief compared to other anxiolytics [14].

After a single dose of alprazolam, it takes about 1.8 hours to reach peak plasma concentration (*C*_max_). The sublingual dosage form takes relatively longer time [15]. The amount of drug ingested and peak plasma concentration are proportional. The elimination half-life (*t*_1/2_) is from 10 to 18 hours after a single oral dose [16]. The mean absolute bioavailability of oral alprazolam was found to be 92% compared to that of intravenous alprazolam. The onset of alprazolam-induced sedation was reported to occur more rapidly than oral administration after intravenous administration. However, the volume of distribution is estimated to be higher in oral form than in the intravenous form [16]. Coadministration with food does not alter the rate or extent of absorption [15]. There is no apparent difference in pharmacokinetics profile between men and women [17]. In pregnant women, use in the first trimester is associated with increased risk of congenital abnormalities [18] and therefore it is considered as pregnancy category D. The clearance and the half-life for multiple doses are similar to those of single dose administration. In multiple dose administration, the steady state concentration is proportional to daily dose and is similar to that found by single dose [16]. Peak plasma levels are higher in elderly population and clearance is reduced [19, 20].

Alprazolam or benzodiazepines in general mainly exert their function through gamma aminobutyric acid (GABA_A_) receptor, which consists of three subunits: *α* (alpha), *β* (beta), and *γ* (gamma). Mainly, *α*_1_ subunit is associated with the sedative and amnestic function of benzodiazepines whereas *α*_2_ is associated with the anxiolytic effect [21]. *α*_1_ subunit is present mainly in the cerebellum and *α*_2_ can be found in the hippocampus, striatum, and spinal cord (reviewed in [21]).

Alprazolam has long been studied to observe its role in developing abuse potential. Inhaled and oral dosage form of alprazolam could increase the abuse potential of the drug in subjects with histories of drug abuse [22]. Alprazolam is scheduled as a psychotropic substance by the WHO and is also reported to be the most used benzodiazepine with alcohol for abuse purposes in Drug Abuse Warning Network (DAWN) report [23].

Alprazolam has been reported to develop aggression-like behavior upon chronic use (reviewed by [24]) but in case of patients with dementia alprazolam decreases agitation with a significant improvement in symptoms on clinical global impression scale [25]. Alprazolam has also been reported to have the lowest risk associated with postprescription nonvertebral fractures in elderly patients [26] and the premedication of alprazolam with melatonin has been reported to improve anxiolysis in addition to having an effect on sedation score and amnesia [27].

However, the cognitive side effects of alprazolam have received the greatest attention in the majority of the studies. In general, benzodiazepines have been reported to produce general CNS side effects and cognitive impairment. Sedation, reduced alertness, drowsiness, sleepiness, confusion, and headache constitute the general side effects whereas poor attention and anterograde amnesia are thought to be the cognitive impairment [28]. Verster et al. [29] have demonstrated the acute effects of 1 mg alprazolam, which impairs psychomotor performance and special cognitive skills required for daily activities like driving ability. Furthermore, Leufkens et al. [30] have added on the subject of acute effect of immediate and extended release formulation of alprazolam in healthy volunteers on the abovementioned study parameters. And thus the majority of the studies conducted with alprazolam focused on the acute challenge posed immediately upon alprazolam administration (reviewed in [21]). These studies clearly show that acute challenge with alprazolam deteriorates specific aspects of cognitive function. However, it has been reported that some of the benzodiazepine-induced side effects improve upon time if not completely eliminated [31]. It is still unresolved whether the cognitive domains impaired by immediate administration of alprazolam remained the same upon chronic use. Those few studies conducted with chronic uptake of alprazolam have focused mainly on psychomotor performance and sleep activity and have drawn different conclusions (reviewed in [21]). Impairment of psychomotor performance was found when alprazolam was administered at a high dose for 3 weeks [32] or 0.25 mg t.i.d. for one week [33]. On the other hand, other studies found no impairing effects when administering a total of 4 mg drug for 4 days [34], 0.5 mg once daily [35], or 0.25 mg once daily [36] for one week. As for two-week period studies, it was reported that alprazolam 0.25 mg t.i.d. did not impair psychomotor performance [14]; however, another study claimed it to be improved upon repeated use of 0.125 mg twice daily [37]. Tests used in these studies also vary in nature and none of these studies reported possible effect upon chronic administration of alprazolam on attention, psychomotor analysis, and memory together on study subjects.

Therefore, we explored the possible effects on memory, attention, and psychomotor performance in healthy male volunteers who were kept on alprazolam for two weeks. The outcomes were measured using Cambridge Neuropsychological Test Automated Battery (CANTAB) software. Assessment of cognitive functions with CANTAB has been proved to be superior to other traditional psychometric tests because of its language and culture independency, higher subjective compliance, and standardized tests. The validity of the CANTAB tests has also been assessed by various researchers making it a preferred choice to study cognitive functions. One study ran a preliminary validity test to assess the execution function in patients with schizophrenia and bipolar disorder where it compared the results with Computerized Neurological Test (CNT) [38]. Another study conducted a comparison of the CANTAB tests with “traditional” neuropsychological testing instruments. The authors in [39] reported a modest association with traditional neuropsychological test measures. We assumed that the battery of CANTAB tests selected is sensitive enough to detect any impairment or improvement that might occur over the treatment period.

## 2. Methods and Assessment

### 2.1. Participants

The present study was conducted on 26 healthy male volunteers. All the volunteers were recruited from the University of Asia Pacific, Bangladesh. All were conversant with English to carry out the instructions given on the screen during the test. The healthy volunteers were randomly assigned to two groups. Among them, 13 were treated with alprazolam and the remaining 13 were considered as placebo (control) group. The range of age varied between 20 and 23 years. Prior to the study, written consent was obtained from all the participating volunteers. To determine the eligibility of the study volunteers as healthy individuals, they were provided with medical health questionnaires. Participants were asked to provide their medical and psychiatric history for the last six months before taking part in the study. If the participants were not able to provide sufficient information, they were not recruited in the study. Intelligence quotient (IQ) was measured by National Adult Reading Scale as mentioned previously [40]. None of the volunteers were habitual smokers. They did not have any history of alcohol intake. It was also ensured that study subjects did not consume any caffeine before 12 hours upon completion of the test. Institutional ethical approval was obtained (ZSC201401). The study strictly followed the International Conference of Harmonization (ICH) for Good Clinical Practice (GCP) and it was conducted in compliance with the Declaration of Helsinki and its further amendments.

### 2.2. Treatment and Design

**Figure 1:**
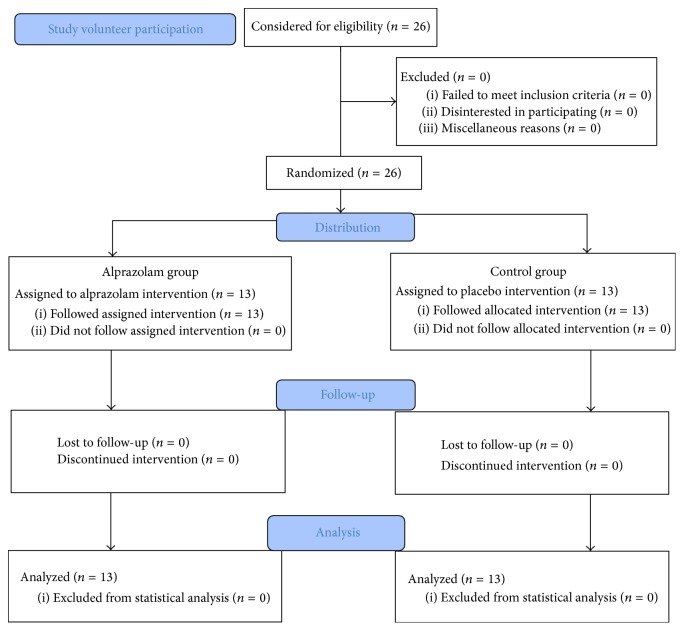
CONSORT 2010 Flow Diagram.

The duration of the study was two weeks. Volunteers were divided into two groups randomly (Figure 1). A dose of 0.5 mg was selected for this particular study. Although the usual dose ranges from 0.25 to 0.5 mg three times daily, a low dose was selected to avoid subsequent dose tapering after completion of the study. All recruited subjects were students and multiple dosing was assumed to lead to potential dose missing. One group took local brand of alprazolam (containing 0.5 mg alprazolam) every night between 9 p.m. and 10 p.m. for two weeks. Nighttime dosing was selected to avoid possible occurrence of drowsiness to be considered as a side effect of the treatment in only one group, which may occur if given at daytime. The control group was assigned to take placebo following the same time length and pattern. The different outlooks like texture, shape, size, and color for both alprazolam and placebo were the same. Besides, as mentioned above, a nighttime dosing schedule was selected to minimize participant's own intuition about the treatment. The volunteers were briefed thoroughly so that they have a clear idea about the tests before they start them. Before the initiation of the first dose administering with either alprazolam or placebo, the condition of memory, attentiveness, and psychomotor performance was measured to determine the baseline data. After two weeks, the subjects performed the tests again just after 8 hours of their last dose. The sequence of selected tests was maintained to be the same for all the volunteers. It was also ensured that the volunteers do not know whether they are taking alprazolam or placebo. The groups were revealed only after the last test was done for the last study volunteer. Volunteers had the option to contact the study center in case of any emergency during the test. Constant communication was maintained with all the participants throughout the study to keep track of the intake of the recommended product.

### 2.3. Assessment

CANTAB was implied to assess the memory, attention, and psychomotor function of the volunteers. Considering instruction from the CANTAB developers (product manual and web resource) and our previous investigations [41] and based on the study purpose, a battery of four different neuropsychological tests were selected for memory, attention, and psychomotor performance. Paired Associates Learning (PAL) and Delayed Matching to Sample (DMS) were selected to study effects on visual memory. Rapid Visual Information Processing (RVP) and Choice Reaction Time (CRT) were selected to observe the possible effect of alprazolam on attention and psychomotor performance, respectively. This computerized platform is now widely used for assessing cognitive function, memory, and attention. Our group has demonstrated the validity of these subtests on healthy volunteers [41, 42]. Similarly, other researchers working on Alzheimer's disease [43] and with ataxia patients [44] have also reported the validity of these subtests.

*Test of Visual Memory*. Test of visual memory was carried out using the following.

*Paired Associates Learning (PAL)*. PAL test is developed to measure visual memory and new learning. It measures memory in an episodic manner, which requires remembering a particular location previously paired with an object.

One or more boxes with different patterns inside were displayed to the participants. There were eight different positions on the screen in which the boxes can appear. The boxes appeared in random orders on those positions but only one box at a time. The patterns were then displayed in the middle part of the screen, one at a time. Then, study subjects had to identify the exact position of the box in which the pattern was present. This test has gradual pattern of progress so that the number of boxes with patterns increases as subjects complete the previous stages. Clinical mode was selected for this study, which has eight stages. Each stage should be completed by a maximum of ten attempts. Evaluation is based on the following:

- Total errors adjusted (total errors committed in all stages and adjustment for each stage not attempted because of prior failure).
- Mean error to success (mean errors done before successful completion of a stage).
- Mean trial to success (total trials needed to locate all patterns accurately).
- Memory score on the first trial.

*Delayed Matching to Sample (DMS)*. DMS is the test for determination of visual memory. The memory process is examined in a nonverbal manner in this test. A decline in perception or attention may affect the outcome of the study. The systemic time interval and sensitivity in precision of patterns make this test more robust to study visual memory.

A complex visual pattern is presented to the subject for 4.5 seconds, which is considered as the sample. With or without a brief delay, four similar patterns are displayed to the subject. The perfect pattern-match had to be identified by the participant. Sample and choice making patterns may be shown simultaneously or after a delay of 0, 4, or 12 seconds. A single test consists of 43 trials among which the first three are not evaluated. A subject can make a maximum of four choices to match the pattern in each trial. More choices led to an increase in choice latency. Evaluation is based on the following:

- Probability of error following error.
- Probability of error following correct response.
- Correct total.
- Correct simultaneous.
- Correct for 0 s delay (delay between presentation of sample pattern and choice pattern).
- Correct for 4 s delay (delay between presentation of sample pattern and choice pattern).
- Correct for 12 s delay (delay between presentation of sample pattern and choice pattern).
- Correct for all delay.
- Mean latency (average time needed to respond with accurate response).

*Test of Attention*. Attention is assessed using the following.

*Rapid Visual Information Processing (RVP)*. RVP is more focused towards the assessment of attention. RVP examines the attention that is visual and sustained. It also measures continuous performance. In this test, different single digits (ranging from 2 to 9) appear in a box placed in the middle of the screen. The digits are shown in a pseudo random order, one at a time with a rate of 100 digits appearing per minute. During the test, subjects had to identify a particular sequence of numbers (2, 4, 6; 3, 5, 7; or 4, 6, 8) displayed at the upper right side of the box, from the randomly appearing single digits in the box. Whenever a subject identifies the target sequence from the random presentation of single numbers, he/she had to register the response by using the press pad. Successful registration was counted as hit. Pressing the pad irrespective of the target sequence was counted as miss. A single test consisted of 7 attempts in total. Among them, the first four attempts were not evaluated. The target sequences appeared 27 times in the latter three attempts. Evaluation is based on the following:

(a) RVP A′: probability for the identification of the target sequence.
(b) RVP B′′: probability to depress the press pad irrespective of the occurrence of target sequence.
(c) RVP total hits: the number of occasions upon which the target sequence was correctly identified.

*Test of Psychomotor Skills*. Psychomotor skills are assessed using the following.

*Choice Reaction Time (CRT)*. CRT is a reaction time test. This test follows 2-Choice Reaction Time test where speed of response provides the evaluation. This test measures alertness and motor speed. Two conceivable stimuli and responses were introduced to the study subjects. Stimulus was displayed in an “arrow shape” which appeared either on the left or on the right side of the computer screen. The study subject was supposed to follow arrow direction to press the corresponding left or right “press pad.” The duration of response limit was 3.1 seconds. A single test consisted of three attempts among which the first one was not evaluated. The latter two attempts each had 50 trials. The average prestimulus delay in both attempts was around 1.1 seconds. Evaluation was based on mean latency of response.

### 2.4. Statistical Analysis

Results were analyzed independently for each test. To find the difference between alprazolam and placebo group, we checked normality assumption and employed statistical tests that are appropriate. For each measure, performance under the drug condition was compared with that at baseline using a 2 (time; baseline, after two weeks) × 2 (treatment; placebo, alprazolam) mixed model ANOVA with repeated measures by using IBM Statistics 21 to find the effect of alprazolam over the period of two weeks. Chi-square test was performed for demographic data between groups. *p* < 0.05 was considered statistically significant.

## 3. Result

### 3.1. Demographic Data

Randomly recruited volunteers in either group did not vary significantly in their age and estimated IQ (*p* > 0.05). The age (mean ± standard deviation) of the volunteers was 20.92 ± 0.95 and 21.00 ± 1.00 years for alprazolam and placebo group, respectively, with *p* = 0.891. The IQ (mean ± standard deviation) of the volunteers was 113.46 ± 11.25 and 114.23 ± 9.97 for alprazolam and placebo group, respectively, with *p* = 0.912.

### 3.2. Test of Visual Memory

*PAL*. Mixed model repeated measures ANOVA with 2 levels of group and 2 levels of time shows that one out of four tests of PAL had significant main effect of treatment for two weeks with *F*(1,24) = 14.45, *p* = 0.001, *η* ^2^ = 0.376 for PAL mean trial to success; *F*(1,24) = 0.629, *p* = 0.435, *η* ^2^ = 0.026 for PAL total errors adjusted; *F*(1,24) = 0.740, *p* = 0.398, *η* ^2^ = 0.030 for PAL mean error to success; and *F*(1,24) = 3.396, *p* = 0.078, *η* ^2^ = 0.124 for PAL memory score on the first trial. There was significant interaction between treatment for two weeks and PAL mean trial to success with *F*(1,24) = 37.47, *p* < 0.001, *η* ^2^ = 0.610 indicating that alprazolam impaired PAL mean trial to success significantly over time. Since all the subjects had the value of 4 for “PAL stages completed” at both time points, analysis of this parameter was not possible and was therefore excluded from analysis (Supplementary Table  1, in Supplementary Material available online at http://dx.doi.org/10.1155/2016/3730940).

*DMS*. Mixed model repeated measures ANOVA with 2 levels of group and 2 levels of time shows that three out of the nine tests of DMS had significant main effect of treatment for two weeks with *F*(1,24) = 7.708, *p* = 0.010, *η* ^2^ = 0.243 for DMS correct 0 s delay; *F*(1,24) = 7.078, *p* = 0.014, *η* ^2^ = 0.228 for DMS correct 4 s delay; *F*(1,24) = 5.237, *p* = 0.031, *η* ^2^ = 0.179 for DMS correct all delay; *F*(1,24) = 0.125, *p* = 0.727, *η* ^2^ = 0.005 for DMS probability of error following error; *F*(1,24) = 0.323, *p* = 0.575, *η* ^2^ = 0.013 for DMS probability of error following correct response; *F*(1,24) = 3.743, *p* = 0.065, *η* ^2^ = 0.135 for DMS correct total; *F*(1,24) = 0.000, *p* = 1.000, *η* ^2^ = 0.000 for DMS correct simultaneous; *F*(1,24) = 0.064, *p* = 0.803, *η* ^2^ = 0.003 for DMS correct 12 s delay; and *F*(1,24) = 1.721, *p* = 0.202, *η* ^2^ = 0.067 for DMS mean latency. There was significant interaction between treatment for two weeks and DMS correct 0 s delay with *F*(1,24) = 1.046, *p* = 0.010, *η* ^2^ = 0.243; between treatment for two weeks and DMS correct 4 s delay with *F*(1,24) = 0.020, *p* = 0.890, *η* ^2^ = 0.001; and between treatment for two weeks and DMS correct all delay with *F*(1,24) = 1.061, *p* = 0.313, *η* ^2^ = 0.042 indicating that alprazolam impaired DMS correct 0 s delay, DMS correct 4 s delay, and DMS correct all delay significantly over time (Supplementary Table  1).

### 3.3. Test of Attention

*RVP*. Mixed model repeated measures ANOVA with 2 levels of group and 2 levels of time shows that one of the tests of RVP, RVP total hits, had significant main effect of treatment for two weeks with *F*(1,24) = 21.608, *p* = 0.000, *η* ^2^ = 0.474 but there was no such effect (*p* > 0.05) observed for RVP A and RVP B with *F*(1,24) = 0.990, *p* = 0.330, *η* ^2^ = 0.040 and *F*(1,24) = 1.600, *p* = 0.218, *η* ^2^ = 0.062, respectively. However, significant interaction between treatment for two weeks and group was not found with *F*(1,24) = 0.031, *p* = 0.861, *η* ^2^ = 0.001 indicating that error terms are so high that the interaction for RVP total hits could not be calculated over time (Supplementary Table  2).

### 3.4. Test of Psychomotor Performance

*CRT*. Mixed model repeated measures ANOVA with 2 levels of group and 2 levels of time shows that there is no main effect of treatment for two weeks with *F*(1,24) = 1.425, *p* = 0.244, *η* ^2^ = 0.056 for CRT mean latency indicating that alprazolam treatment neither impaired nor improved psychomotor performance (Supplementary Table  3).

## 4. Discussion

In the present study, we observed the effect of 0.5 mg alprazolam daily on healthy volunteers for two weeks. After observing individual performance in PAL test, it was revealed that the subjects who performed poorly before initiation of the therapy made fewer errors after alprazolam treatment whereas subjects who made fewer errors took more attempts to complete the test after the treatment. This seemingly opposing effect can be interpreted as increased focus and attention of the subjects who failed more on the first occasion. It has been suggested that subjective performance will not be impaired due to drug's effect if the cognitive and performance test duration is not considerably long (reviewed in [21]). As the Paired Associates Learning test required 10–15 minutes to complete in either group before and after treatment, we assume that the subjects who previously failed more tended to show more attention than others who performed well before. We also note that the amount of drug intake might also be low that it did not produce any effect on the test parameters after two weeks. Higher doses might yield results showing impairment in Paired Associates Learning in these parameters.

Analysis of DMS test showed that the probability of making an error either following error or following correct response did not change significantly (*p* > 0.05) over two weeks in alprazolam group. Total correct responses when the choice patterns were present simultaneously with the sample pattern were also found to be not different between alprazolam and placebo group (*p* > 0.05). However, a statistically significant difference (*p* < 0.05) was observed in alprazolam group over two weeks of treatment, when there is a delay (0 and 4 seconds) in presentation of the choice pattern. Similarly, when all correct responses were summed up for all delay situations, correct all delay, we also found a significant difference (*p* < 0.05) in alprazolam group over two weeks of treatment. However, total correct responses in overall test increased slightly in both alprazolam and placebo group but such increment was not significantly different. Given that the dose administered was low, high dose might result in significantly fewer total correct responses. Previous studies have confirmed that immediate and delayed learning are affected upon acute alprazolam challenge [45] and it is evident from our study that alprazolam group did not perform equally compared to placebo after two weeks of treatment. This implies that learning was impaired upon long-term alprazolam administration. On the contrary, some studies with chronic administration of alprazolam have found no significant defect on memory as reviewed elsewhere [21]. These studies used immediate and delayed word recalls and picture recognition tests, which are different from CANTAB's DMS test. Since the outcome measurements in CANTAB were collected through software and in the units of milliseconds, the impairment with alprazolam intake was observable in our study. Over two weeks of treatment, the mean latency of matching the sample decreases sharply but not significantly (*p* > 0.05) in alprazolam group. Similar but less prominent trend was also observed in placebo group. This indicates that the subjects tended to match the sample quickly but in the process make less correct matching due to alprazolam intake.

Overall measurement of attention in RVP showed that alprazolam has a significant effect. Probability of hitting the target sequence, RVP A′, and the probability of pressing the touch pad irrespective of target sequence, RVP B′′, were different in alprazolam group over two weeks of treatment. Total targets successfully identified, RVP total hits, increased significantly (*p* < 0.05) in alprazolam group, which indicates that chronic alprazolam ingestion at least at a dose of 0.5 mg daily does not affect attention. After acute alprazolam challenge, attention is commonly affected. Our study shows that at a low dose attention is not affected when the drug is administered chronically. This is in accordance with previous study reporting that small and repeated dosing of alprazolam produced less pronounced behavioral and adverse side effects [34].

We did not find any significant difference in the mean latency of reaction time (milliseconds) in alprazolam group over two weeks of treatment. Both alprazolam and placebo groups showed that the mean latency of reaction time was decreased but not significantly (*p* > 0.05). This is in accordance with the findings of previous studies [34–36, 46] where chronic ingestion of alprazolam did not affect psychomotor performance.

The fact that mean choice latency for DMS and RVP test decreased after alprazolam treatment indicates increment of fine motor controls, which may result from decreased activity of GABA_A_ mediated inhibition or increased excitatory activity of glutamatergic system. It has been proposed that chronic increased GABA receptor mediated inhibition by benzodiazepines may result in increased sensitivity of glutamatergic system, the main excitatory system of the brain [47]. There are studies in animals to support this hypothesis. In a study by Steppuhn and Turski [48], it was demonstrated that mice develop benzodiazepine withdrawal symptoms after chronic treatment, which consists of an initial silent phase and then an active phase characterized by increased anxiety, muscle rigidity, and seizure activity. Administration of N-methyl-D-aspartate (NMDA) receptor antagonist prevented the development of withdrawal symptom in the active phase and administration of *α*-amino-3-hydroxy-5-methylisoxazole-4-propionic acid (AMPA) receptor antagonist in the silent phase prevented subsequent development of withdrawal symptoms of active phase. So far, there are no such studies reported for benzodiazepines withdrawal in humans but it is conceivable that this system becomes more sensitive upon chronic benzodiazepines treatment.

There have been no studies measuring glutamate concentration during benzodiazepine intake in human to the best of our knowledge. Acute alcohol withdrawal increases glutamate concentration and the glutamatergic system becomes sensitized [49]. Hyperactive glutamatergic system can cause damage to superior cortical activity [50], which may also result in chronic benzodiazepine users. Future studies could be directed to observe the occurrence of hyperactive behavior upon chronic benzodiazepine intake along with glutamate concentration to find a correlation between glutamate and hyperactivity.

**Figure 2:**
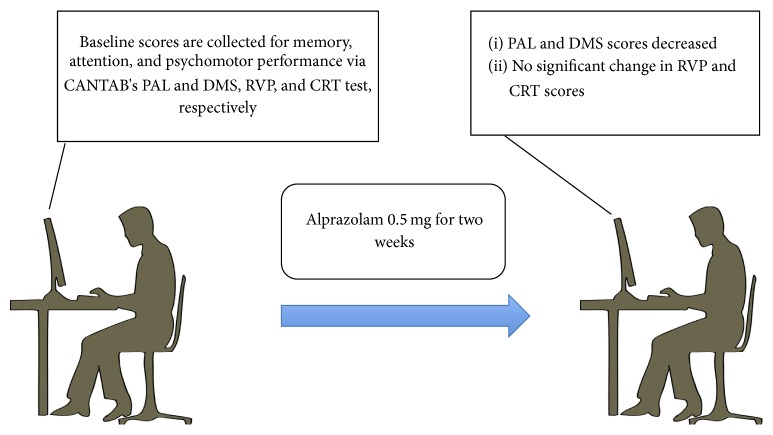
Effect of 0.5 mg alprazolam daily on healthy male volunteers for two weeks. Baseline data are collected for CANTAB's PAL, DMS, RVP, and CRT tests. Then, subjects took 0.5 mg of alprazolam daily for two weeks. After two weeks, PAL and DMS score decreased compared to controls. RVP and CRT scores were unaffected.

Our study indicates that chronic administration of alprazolam intake does not affect psychomotor performance and attention but affects memory performance of healthy volunteers (Figure 2). In a meta-analysis with patients kept on long-term benzodiazepines, it was reported that patients develop certain kinds of cognitive impairment upon withdrawal and during follow-up those impairments remained [51]. These include sensory processing, verbal memory, speed of processing, motor performance, working memory, and verbal speed. We failed to recapitulate motor performance defect in the current study possibly because of the low dose used. Overall, long-term benzodiazepine users may not be in their full cognitive state upon withdrawal. The mechanism of benzodiazepine-induced cognitive effect upon withdrawal and during treatment is not clear. Given that the mechanism of such effects is independent of whether patients have mood disorder or not, the cognitive impairment might be of the same amplitude. However, since mood disorder patients have inherent alternation in brain function, the outcome of the result may vary quite dramatically from that of not having any disorders.

Utilization of CANTAB software to conduct the study yielded more accurate and reproducible results. However, 0.5 mg once daily dose is relatively low compared to standard alprazolam requirement for anxiety relief and nighttime dosing schedule does not mimic actual practice of drug prescription and the sample size was also not large enough. Because of the delay between last dose of the drug and testing of cognitive function, the obtained cognitive scores might not represent the scenario where the drug was at its peak plasma concentration. Inclusion of patients group who are kept on alprazolam treatment for future study is suggested. Although alprazolam has not been reported to have any active metabolite, we also propose to measure alprazolam concentration in subjects over the treatment period in future studies to more appropriately fit the pharmacokinetic and pharmacodynamic profile of this drug.

## Supplementary Material

Alprazolam is used as an anxiolytic drug for generalized anxiety disorder and it has been reported to produce sedation and anterograde amnesia. In the current study, the Cambridge Neuropsychological Test Automated Battery (CANTAB) software was used to test the effect of chronic intake of alprazolam in healthy volunteers. The selected testing battery consisted of the Paired Associates Learning (PAL) and Delayed Matching to Sample (DMS) tests for memory, Rapid Visual Information Processing (RVP) for attention, and Choice Reaction Time (CRT) for psychomotor performance. The testing was done twice: before starting the treatment and after the completion of the treatment. In the current investigation, statistically significant impairment of visual memory in one parameter of PAL and three parameters of DMS were found. However, one parameter of RVP was improved and no difference was observed in CRT.
