# Supplementary material for: The Effect of Chronic Alprazolam Intake on Memory, Attention, and Psychomotor Performance in Healthy Human Male Volunteers
Source: Behav Neurol. 2016 Jul 4;2016:3730940. doi: 10.1155/2016/3730940 (PMC4947648; doi:10.1155/2016/3730940)
Supplement: Supplementary file 1 — Alprazolam is used as an anxiolytic drug for generalized anxiety disorder and it has been reported to produce sedation and anterograde amnesia. In the current study, the Cambridge Neuropsychological Test Automated Battery (CANTAB) software was used to test the effect of chronic intake of alprazolam in healthy volunteers. The selected testing battery consisted of the Paired Associates Learning (PAL) and Delayed Matching to Sample (DMS) tests for memory, Rapid Visual Information Processing (RVP) for attention, and Choice Reaction Time (CRT) for psychomotor performance. The testing was done twice: before starting the treatment and after the completion of the treatment. In the current investigation, statistically significant impairment of visual memory in one parameter of PAL and three parameters of DMS were found. However, one parameter of RVP was improved and no difference was observed in CRT. [file 3730940.f1.docx]

**Supplementary file**

**The effect of chronic alprazolam intake on memory, attention and psychomotor performance in healthy human male volunteers**

Zahid Sadek Chowdhury^1^, Mohammed Monzur Morshed^2^, Mohammad Shahriar^1^, Mohiuddin Ahmed Bhuiyan^1^, Sardar Mohd.Ashraful Islam^1^, Muhammad Shahdaat Bin Sayeed ^1,3^*

^1^Department of Pharmacy, University of Asia Pacific, Dhaka-1209, Bangladesh

^2^Department of Biochemistry and Molecular Biology, University of Dhaka, Dhaka-1000, Bangladesh

^3^Department of Clinical Pharmacy and Pharmacology, University of Dhaka, Dhaka-1000, Bangladesh

*For Correspondence

**Muhammad Shahdaat Bin Sayeed**

Department of Clinical Pharmacy and Pharmacology

University of Dhaka, Dhaka-1000, Bangladesh

Email: shahdaat2013@du.ac.bd , shahdaat2013@yahoo.com

Mob: 08801713459747 Tel: 088029664953 Fax: 088029664950

Web: https://scholar.google.com/citations?user=LobC4uIAAAAJ&hl=en

| Neuropsychological tests | | | Alprazolam group (n=13) | | Control group (n=13) | |
| --- | --- | --- | --- | --- | --- | --- |
|  |  |  | Baseline | After two week | Baseline | After two week |
| Visual Memory | PAL (Paired Associates Learning) | Total errors adjusted* | 3.62±3.01 | 3.38±3.18 | 3.85±3.51 | 2.85±2.79 |
|  |  | Mean error to success | 0.45±0.38 | 0.42±0.40 | 0.49±0.43 | 0.36±0.35 |
|  |  | Mean trial to success | 1.45±0.38 | 1.26±0.12 | 1.27±0.17 | 0.48±0.44 |
|  |  | Memory score on first trial | 12.46±5.03 | 14.0±4.74 | 13.54±5.08 | 15.38±4.35 |
|  | DMS (Delayed Matching to Sample) | probability of error following error | 0.12±0.08 | 0.11±0.04 | 0.09±0.03 | 0.1±0.03 |
|  |  | probability of error following correct response | 0.15±0.12 | 0.13±0.05 | 0.11±0.04 | 0.11±0.03 |
|  |  | Correct Total | 34.08±4.63 | 35.0±2.12 | 33.31±3.82 | 35.46±2.47 |
|  |  | Correct simultaneous | 9.08±1.55 | 9.15±0.80 | 8.92±0.76 | 8.85±0.69 |
|  |  | Correct for 0s delay* | 8.54±1.33 | 9.0±0.91 | 8.15±0.07 | 9.15±0.8 |
|  |  | Correct for 4s delay* | 8.31±1.32 | 9.0±1.22 | 8.23±1.17 | 9.0±1.0 |
|  |  | Correct for 12s delay | 8.15±1.28 | 7.85±1.21 | 8.0±1.41 | 8.46±0.88 |
|  |  | Correct for All delay* | 25.0±3.37 | 25.85±1.99 | 24.38±3.28 | 26.62±2.18 |
|  |  | Mean latency in milliseconds | 3915.72±1189.61 | 3525.63±794.41 | 4026.92±1076.47 | 3941.15±1159.83 |

**Supplementary Table 1: The effect of chronic alprazolam intake on visual memory**

* indicates p<0.05, The values are expressed as Mean ± Standard Deviation

**Supplementary Table 2: The effect of chronic alprazolam intake on attention**

| Neuropsychological tests | | | Alprazolam group (n=13) | | Control group (n=13) | |
| --- | --- | --- | --- | --- | --- | --- |
|  |  |  | Baseline | After two week | Baseline | After two week |
| Attention | RVP  (Rapid Visual Information Processing) | RVP A [p(hit)] | 0.89±0.09 | 0.90±0.06 | 0.90±0.09 | 0.92±0.06 |
|  |  | RVP B" [p(hit)+p(miss)] | 0.95±0.04 | 0.96±0.03 | 0.95±0.06 | 0.95±0.04 |
|  |  | RVP Total Hits* | 15.77±3.77 | 18.92±4.70 | 15.92±5.47 | 18.85±5.34 |

* indicates p<0.05, The values are expressed as Mean ± Standard Deviation

**Supplementary Table 3:** **The effect of chronic alprazolam intake on psychomotor performance**

| Neuropsychological tests | | | Alprazolam group (n=13) | | Control group (n=13) | |
| --- | --- | --- | --- | --- | --- | --- |
|  |  |  | Baseline | After two week | Baseline | After two week |
| Psychomotor performance | CRT (Choice reaction Time) | CRT (Mean latency in milliseconds) | 360.03±49.56 | 349.94±56.69 | 382.31±32.44 | 373.85±31.50 |

* indicates p<0.05, The values are expressed as Mean ± Standard Deviation
